# Immune Responses to Epidermal Growth Factor Receptor (EGFR) and Their Application for Cancer Treatment

**DOI:** 10.3389/fphar.2016.00405

**Published:** 2016-10-26

**Authors:** Tetsuro Sasada, Koichi Azuma, Junya Ohtake, Yuki Fujimoto

**Affiliations:** ^1^Cancer Vaccine Center, Kanagawa Cancer Center Research Institute, YokohamaJapan; ^2^Division of Respirology, Neurology, and Rheumatology, Department of Internal Medicine, Kurume University School of Medicine, KurumeJapan

**Keywords:** EGFR, mutation, cellular immune response, humoral immune response, PD-L1

## Abstract

Epidermal growth factor receptor (EGFR) is a prototypic cell-surface receptor belonging to the ErbB/HER onocogene family. Overexpression or somatic mutations of EGFR have been reported to play an important role in tumorigenesis in various types of epithelial cancers. Therefore, targeting of EGFR with specific blocking antibodies or inhibitors have been developing for treatment for EGFR-associated tumors. Immune responses to HER2, another molecule of the ErbB/HER onocogene family, have been well studied, but only limited information on the immune responses to EGFR in cancer has been currently available. In this review, we have summarized the available data and discussed potential clinical importance of the anti-EGFR immune responses and EGFR-mediated immune regulation in cancer. Several lines of evidence suggest that cellular and humoral immune responses to EGFR might be useful as a marker and/or target for cancer therapy against EGFR-associated tumors. In addition, recent studies suggest the critical roles of EGFR-mediated signaling in regulation of expression of an immune checkpoint molecule, programmed death-ligand 1 (PD-L1) in tumor cells. Further studies are warranted to clarify the impact of the anti-EGFR immune responses and EGFR-mediated immunomodulation for clinical application for cancer treatment.

## Introduction

Epidermal growth factor receptor (EGFR), a prototypic cell-surface receptor belonging to the ErbB/HER onocogene family, has been known to play an important role in the regulation of cell proliferation, differentiation, and migration (Baselga and Arteaga, 2005; Tebbutt et al., 2013). In addition, oncogenic somatic mutations in specific sites of the EGFR gene, such as EGFR variant III (EGFRvIII) in glioblastoma multiforme (GBM) and activating EGFR mutations in non-small cell lung cancer (NSCLC), have been reported to be closely associated with tumorigenesis (Guo et al., 2015; Tan et al., 2015). Since EGFR is overexpressed in various types of epithelial cancers, including pancreatic, colorectal, breast, and lung cancer, it has been suggested that EGFR might be an appropriate target for cancer therapy. Indeed, EGFR-associated tumors have been targeted by specific blocking antibodies or inhibitors for cancer treatment. In addition, personalized cancer treatments targeting the oncogenic EGFR mutations are currently developing.

Immune responses to HER2, another molecule of the ErbB/HER onocogene family, have been well studied (Chapman et al., 2008; Knutson et al., 2016; Tabuchi et al., 2016), but only limited information on the immune responses to EGFR in cancer has been currently available. We have been studying cellular and humoral immune responses specific to EGFR (Yamada et al., 2013; Azuma et al., 2014a), as well as modulatory effect of EGFR-mediated signaling in expression of an immune checkpoint molecule, programmed death-ligand 1 (PD-L1) (Azuma et al., 2014b; Ota et al., 2015). In the current review, we have summarized the currently available data and discussed potential clinical importance of anti-EGFR immune responses and EGFR-mediated immune regulation.

## Cellular Immune Responses to Wild Type EGFR

Cellular immune responses specific to wild-type EGFR have been studied in patients with several types of cancer, including NCSLC and head and neck squamous cell carcinoma (HNSCC). For example, Shomura et al. (2004a) identified three HLA-A24-restricted epitopes derived from wild-type EGFR at positions 54–62, 124–132, and 800–809, which were able to induce both specific cellular and humoral immune responses in most of NCSLC patients. In addition, the same group also reported two HLA-A2-restricted epitopes derived from wild-type EGFR at positions 479–488 and 1138–1147, both of which were able to induce both specific cellular and humoral immune responses in NCSLC patients (Shomura et al., 2004b). Interestingly, Schuler et al. (2011) demonstrated that the frequency of cytotoxic T cell (CTL) specific to these two HLA-A2-restricted epitopes in peripheral blood of HNSCC patients correlated significantly with EGFR expression in tumor tissues. HNSCC patients with elevated EGFR expression had a significantly higher frequency of EGFR-specific CTL than those with lower EGFR expression or normal individuals. This finding suggested that EGFR expressed on tumor cells might induce a specific cellular immune response *in vivo*. We have used one of the HLA-A24-restricted epitopes, EGFR_800-809_, as a candidate peptide for personalized peptide vaccination, in which vaccine antigens are selected and administered based on the pre-existing host immunity before vaccination (Sasada et al., 2014). Recent clinical trials of personalized peptide vaccination have demonstrated significantly prolonged overall survival in patients with advanced cancers, including prostate and bladder cancer, through induction of antigen-specific T cells (Sasada et al., 2014; Noguchi et al., 2016; Yoshimura et al., 2016).

Andrade Filho et al. identified another immunogenic HLA-A2-restricted epitope, EGFR_853-861_, derived from wild-type EGFR, and showed that EGFR_853-861_-specific T cells were increased in the peripheral circulation of HNSCC patients (Andrade Filho et al., 2010). Notably, they demonstrated that recognition of HNSCC cells by EGFR_853-861_-specific T cells was enhanced by incubation of tumor cells with the EGFR-blocking monoclonal antibody (mAb) cetuximab, which might facilitate antigen presentation through enhanced internalization and proteasomal degradation of EGFR (Vincenzi et al., 2008) and STAT-1-induced HLA class I upregulation (Srivastava et al., 2015). These results suggest that EGFR-specific T cells in cetuximab-treated HNSCC patients may contribute to anti-tumor activity relevant to clinical responses. In addition, they showed that cetuximab-activated NK cells promote DC maturation, which increased EGFR-specific T cell responses through enhanced cross-presentation of EGFR for T cell priming (Srivastava et al., 2013). It may thus be possible that EGFR-specific T cell responses would be useful as a potential biomarker to monitor effects of cetuximab treatment in HNSCC patients.

Kumai et al. (2013) identified a novel promiscuous helper T cell epitope, EGFR_875-889_, which is restricted by several different HLA class II types, including HLA-DR4, HLA-DR15, and HLA-DR53. EGFR_875-889_-specific CD4 T cells can be detected in the peripheral blood of HNSCC patients, and was shown to react against tumor cells expressing EGFR as well as other ErbB/HER family members (HER-2, HER-3) and c-kit, which have homologous peptide sequences. Interestingly, treatment of tumor cells with EGFR tyrosine kinase inhibitors (EGFR-TKI) was shown to upregulate HLA-DR expression on tumor cells and enhance recognition by EGFR_875-889_-specific CD4 T cells.

Although direct therapeutic targeting of EGFR by the blocking mAbs and specific inhibitors have been extensively studied, there have been limited information on therapeutic application of EGFR-specific T cell responses. Clinical trials are warranted to show a feasibility of immunotherapeutic approach targeting EGFR.

## Cellular Immune Responses to Mutated EGFR

Since mutated genes are recognized as foreign by the host immune system, they might elicit stronger immune responses and can be an appropriate target for cancer immunotherapy (Schumacher and Schreiber, 2015). Several studies have been reported regarding immune responses to tumor-specific EGFR mutations, such as EGFRvIII in GBM and EGFR T790M in NSCLC.

EGFR variant III is a tumor specific mutation that is widely expressed in GBM and other neoplasms (Sampson et al., 2008; Guo et al., 2015). This mutation encodes a constitutively active tyrosine kinase that is reported to be associated with tumorigenicity and cellular resistance to chemotherapeutic agents or radiation. EGFRvIII is an in-frame deletion mutation that introduces a novel glycine residue at the fusion junction between distant parts of the wild-type EGFR sequence. This sequence rearrangement has been reported to create a tumor-specific neoepitope that elicit specific cellular and humoral immune responses in GBM patients (Sampson et al., 2008; Guo et al., 2015). As immunotherapeutic approach to GBM that express EGFRvIII, an investigational vaccine (rindopepimut) consisting of the unique EGFRvIII peptide sequence conjugated to keyhole limpet hemocyanin (KLH) has been tested in clinical trials. Multiple phase I and phase II clinical trials demonstrated that the vaccine generated specific cellular and humoral immune responses and tended to show clinical benefits in GBM patients with EGFRvIII mutation (Sampson et al., 2008, 2010; Schuster et al., 2015). Following these promising results, a randomized, double-blind, controlled phase III study named ACT IV, in which rindopepimut plus GM-CSF or KLH alone (control) were administered in combination with standard of care temozolomide, was conducted in newly diagnosed EGFRvIII-positive GBM patients. However, disappointedly the ACT IV study was discontinued in March 2016 based on the recommendation of the independent Data Safety and Monitoring Board that the study was unlikely to meet its primary overall survival endpoint in patients with minimal residual disease (according to the press release of Celldex Therapeutics, Inc.).

**FIGURE 1:**
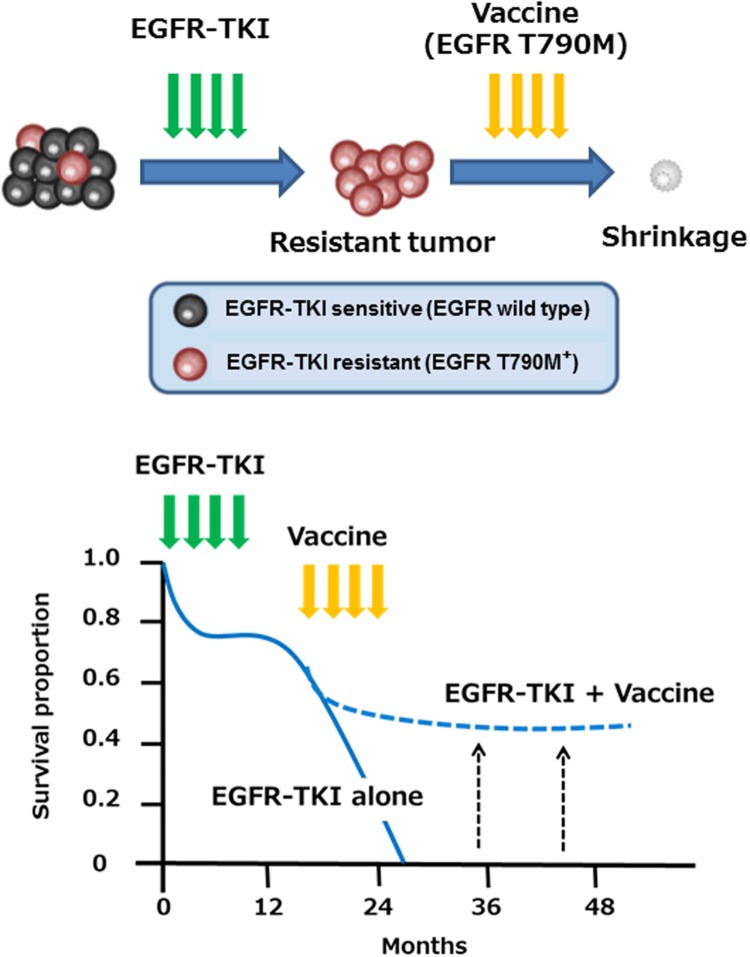
**Immunotherapy with EGFR T790M-derived peptides against NSCLC patients with EGFR T790M mutation.** EGFR T790M-derived immunogenic T cell epitopes might provide a novel immunotherapeutic approach for prevention and/or treatment of EGFR-TKI resistance associated with the EGFR T790M mutation in NSCLC patients.

We and others have reported immune responses to another EGFR mutation, EGFR T790M, which occurs in around 60% of NSCLC patients with acquired resistance to EGFR-TKI, such as gefitinib and erlotinib (Yamada et al., 2013; Ofuji et al., 2015). Whereas most NSCLC patients with EGFR activating mutations found in the exons 19 or 21 of the EGFR gene transiently benefit from treatment with EGFR-TKI, almost all individuals eventually develop resistance to these drugs over time (median of 6–12 months). The secondary T790M mutation has been reported to be associated with resistance to EGFR-TKI by negating the hypersensitivity of activating EGFR mutations (Tan et al., 2015). We have identified two novel HLA-A2-restricted T cell epitopes (MQLMPFGCLL and LIMQLMPFGCL) containing the mutated methionine residue of the EGFR T790M as potential targets for EGFR-TKI-resistant patients (Yamada et al., 2013). The identified EGFR T790M-derived epitopes induced much stronger T cell responses, compared to the epitopes derived from wild-type EGFR. In addition, Ofuji et al. (2015) also reported another HLA-A2-restricted T cell epitope (IMQLMPFGC) derived from EGFR T790M. Of note, a negative correlation between the immune responses to the EGFR-T790M-derived epitopes and the presence of EGFR-T790M mutation was observed in NSCLC patients (Yamada et al., 2013). It may thus be possible that EGFR T790M-specific immune responses might prevent the emergence of tumor cell variants with the T790M mutation in NSCLC patients during EGFR-TKI treatment. Given high immunogenicity in human T cells, the identified T cell epitopes might provide a novel immunotherapeutic approach for prevention and/or treatment of EGFR-TKI resistance associated with the EGFR T790M mutation in NSCLC patients (**Figure 1**). Clinical trials are recommended for development of immunotherapy targeting EGFR T790M.

## Humoral Immune Response to EGFR

Humoral immune responses to HER2 have been extensively studied, and their clinical importance in a variety of cancers has been reported (Chapman et al., 2008; Knutson et al., 2016; Tabuchi et al., 2016). However, there have been limited information on humoral immune response to EGFR in cancer. Pandey et al. (2015) showed that higher antibody levels specific to wild-type EGFR or EGFRvIII, which were associated with immunoglobulin allotypes, were related to improved overall survival in GBM patients. Olsen et al. (2013) reported a negative correlation between autoantibody levels against EGFR in serum and disease free survival in breast cancer patients with relapse or death. These results suggest that stronger immune responses to EGFR might be related to better prognosis in cancer, but further studies remain to elucidate the clinical importance of humoral immune response to EGFR in a variety of cancers.

Humoral immune responses to EGFR in NSCLC patients have not been well studied. Therefore, we investigated the clinical significance of humoral immune responses to EGFR-derived peptides in NSCLC patients receiving EGFR-TKI, gefitinib (Azuma et al., 2014a). Gefitinib has been known to be especially effective in patients with activating EGFR mutations, most of which (about 90%) occur in the tyrosine kinase domain of EGFR located in the exon 19 (deletion such as delE746-A750) or exon 21 (L858R point mutation) (Tan et al., 2015). We measured plasma IgG titers to each of 60 different EGFR-derived 20-mer peptides spanning the entire sequence of EGFR in 42 NSCLC patients receiving gefitinib (Azuma et al., 2014a). We reported that IgG titers against three EGFR-derived peptides, EGFR_481-500_, EGFR_721-740_, and EGFR_741-760_, were significantly higher in patients with the exon 21 mutation. On the other hand, IgG titers against two EGFR-derived peptides, EGFR_841-860_ and EGFR_1001-1020_, were significantly lower and higher, respectively, in patients with deletion in the exon 19. These results suggest that EGFR mutations may be associated with humoral responses to EGFR in NSCLC patients. In addition, multivariate Cox regression analysis showed that IgG responses to EGFR_41-60_, EGFR_61-80_, and EGFR_481-500_ were significantly prognostic for progression-free survival independent of other clinicopathological characteristics, whereas those to the EGFR_41-60_ and EGFR_481-500_ were significantly prognostic for overall survival. Although further study is needed to clarify the mechanisms of the increased IgG responses to these sequences, our results may provide new insight for better understanding of humoral responses to EGFR in NSCLC patients.

## Targeting of EGFR With Chimeric Antigen Receptor (CAR)-Modified T Cells

Chimeric antigen receptor is a synthetic molecule designed to redirect T cells to specific antigens expressed on surface of tumor cells. Adoptive immunotherapy with CAR-engineered T cells show long-term durable remission in B cell malignancies (Maude et al., 2014), but not in solid tumors. Application of this approach to solid tumors requires the identification and selection of target antigens with limited expression on surface of normal cells. Since EGFRvIII mutation that is widely expressed in GBM and other neoplasms has a neoepitope resulting from an in-frame deletion of a part of the extracellular domain of EGFR (Sampson et al., 2008; Guo et al., 2015), it might be an excellent target for CAR-engineered T cell therapy. Recently, Johnson et al. (2015) showed potential efficacy of EGFRvIII-targeted CAR-engineered T cells in *in vitro* cell culture system and *in vivo* mouse models. Similarly, Han et al. (2015) also demonstrated the feasibility of EGFRvIII-targeted CAR-engineered NK cells. A phase I clinical trial of EGFRvIII-targeted CAR-engineered T cells is currently underway for patients with EGFRvIII-positive recurrent GBM.

In contrast, wild-type EGFR has been thought inappropriate for a target molecule of CAR-engineered T cells due to possible deleterious recognition of normal cells, because EGFR is expressed not only tumor cells but also normal cells at physiological levels. However, recent reports demonstrated that affinity of single-chain variable fragment (scFv) of CAR can be tuned to distinguish tumor cells from normal cells based on the disparate density of EGFR expression (Caruso et al., 2015; Liu et al., 2015). Additional studies are required for further pre-clinical evaluation of this novel approach.

## Modulation of PD-L1 Expression by EGFR-Mediated Signaling

Blockade of immune checkpoints with mAbs has recently emerged as a new therapeutic tool in oncology (Postow et al., 2015; Topalian et al., 2016). Programmed cell death 1 (PD1), a type 1 transmembrane protein of the immunoglobulin superfamily, is one of the immune checkpoints expressed on the surface of several types of immune cells, including T cells, B cells, and NK cells. Its ligand, PD-L1, is frequently overexpressed in many types of human cancer. The binding of PD-L1 to PD1 induces apoptosis or exhaustion in activated T cells, and blockade of this interaction has been shown to enhance the antitumor activity of T cells. Recent clinical trials have demonstrated that inhibition of the PD-L1–PD1 interaction with the blocking mAbs, such as nivolumab and pembrolizumab, show promising antitumor effects in patients with various malignancies including NSCLC (Postow et al., 2015; Topalian et al., 2016).

**FIGURE 2:**
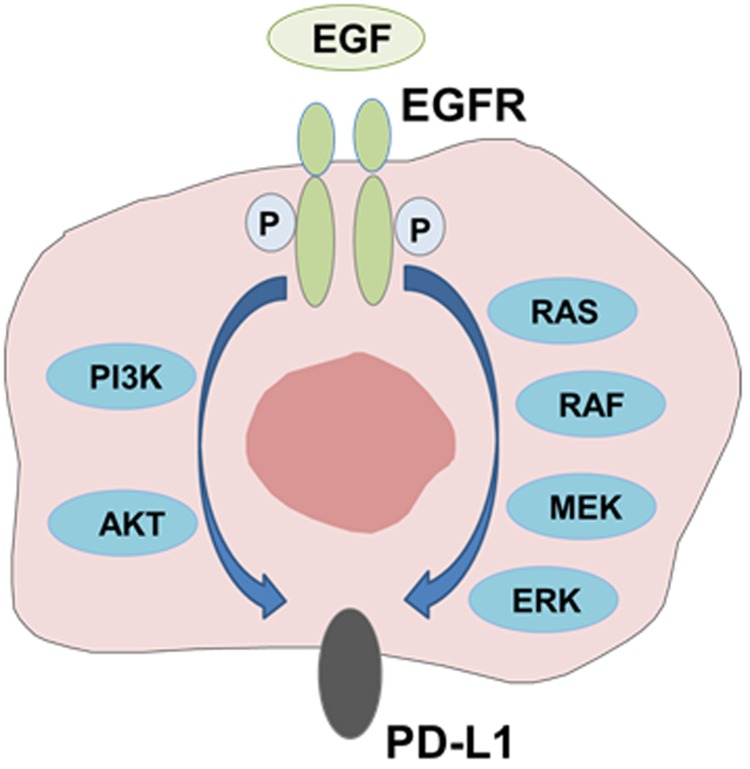
**Modulation of PD-L1 expression by EGFR-mediated signaling.** PD-L1 expression is induced through activation of EGFR signaling pathway mediated by activating EGFR mutations or EGF treatment.

PD-L1 expression has been reported to be driven by some of oncogenic pathways (Topalian et al., 2016). Several studies have reported the association between PD-L1 expression and mutant EGFR mediated signaling. Akbay et al. (2013) showed that mutant EGFR signaling drives increased PD-L1 expression and that blockade of PD1 improved survival of mice in EGFR-driven murine lung tumors. They also demonstrated that forced expression of mutant EGFR induced PD-L1 expression in human bronchial epithelial cell lines, and that EGFR inhibitors reduced PD-L1 expression in NSCLC cell lines with activating EGFR mutations. Similarly, we and others showed that EGFR activation by EGF stimulation or mutant EGFR upregulated PD-L1 expression by activating PI3K-AKT and MEK-ERK signaling pathways in NSCLC cells (Azuma et al., 2014b; Chen et al., 2015; Ota et al., 2015). In addition, Lastwika et al. (2016) demonstrated that active AKT/mTOR signaling mediated by activating EGFR mutation or EGF treatment induced PD-L1 expression in NSCLC cell lines *in vitro* and in mouse models *in vivo*. These results clearly indicate that EGFR downstream signaling pathway mediated by activating EGFR mutations or EGF treatment drives increased PD-L1 expression (**Figure 2**).

In contrast to the studies performed using cell lines and/or *in vivo* mouse models, the correlation between mutant EGFR status and PD-L1 expression in tumor tissues in NSCLC patients seems to be controversial. We examined the association between PD-L1 expression in surgically resected tumor tissues and other clinicopathologic characteristics in 164 NSCLC patients (Azuma et al., 2014b). Multivariate analysis demonstrated that presence of EGFR mutations and adenocarcinoma histology were significantly associated with increased PD-L1 expression independently of other factors. Similarly, D’Incecco et al. (2015) also showed that PD-L1 positivity was significantly associated with adenocarcinoma histology and the presence of EGFR mutations in a cohort of 125 NSCLC patients. Tang et al. (2015) also demonstrated that PD-L1 expression tended to be associated with activating EGFR mutations in 145 advanced lung adenocarcinoma. Interestingly, patients harboring EGFR mutations with higher PD-L1 expression showed more sensitivity to EGFR-TKI probably because of PD-L1 downregulation induced by the EGFR inhibition (D’Incecco et al., 2015; Lin et al., 2015). In contrast, Yang et al. (2014) observed no significant correlation between PD-L1 expression and EGFR, KRAS, BRAF, or ALK mutations in 163 surgically resected stage I lung adenocarcinoma patients. Similarly, Cooper et al. (2015) found no association between PD-L1 expression and EGFR or KRAS mutation status in 678 stage I-III NSCLC patients. Based on these reported studies, the relationship between mutant EGFR status and PD-L1 expression in tumor tissues is inconclusive.

There may be several reasons for such discrepancy. One possible reason may be the heterogeneity of histological cell types (adenocarcinoma vs. squamous cell carcinoma) and/or disease stages (early vs. advanced) in the patients examined (Topalian et al., 2016). Another possible reason may be related to the different protocols and materials (mAbs) employed for immunohistochemical staining (Topalian et al., 2016). In addition, PD-L1 expression has been reported to be driven by not only oncogenic signaling but also adaptive immune responses, such as IFN-γ-mediated stimulation (Topalian et al., 2016). Therefore, more precise and detailed studies are warranted to clarify relationship between mutant EGFR status and PD-L1 expression in tumor tissues. Of note, it has recently been reported that NSCLC patients with activating EGFR mutations show lower objective responsive rates to PD-1/PD-L1 inhibitors (Gainor et al., 2016; Melosky et al., 2016), suggesting the possibility that mutant EGFR status cannot be a satisfactory biomarker for assessing the effects of PD-1/PD-L1 blockade in NSCLC.

## Conclusion

Several lines of recent evidence suggest that immune responses to EGFR might be useful as a marker and/or target for cancer therapy. In particular, since highly immunogenic T cell epitopes have been identified from wild-type and mutant EGFR, their clinical application as a novel immunotherapy might be promising for treatment of EGFR-associated cancer. In addition, recent studies suggest the potential roles of EGFR-mediated signaling in regulation of expression of the checkpoint molecule, PD-L1, in tumor cells, although the correlation between mutant EGFR status and PD-L1 expression in tumor tissues has been inconclusive. Considering notable clinical significance of immune checkpoint blockers, more precise and detailed studies are recommended to clarify the relationship between EGFR status and immune checkpoint expression.

## Author Contributions

TS, KA, JO, and YF were involved in the concept, literature screening, and writing of the article.

## Conflict of Interest Statement

The authors declare that the research was conducted in the absence of any commercial or financial relationships that could be construed as a potential conflict of interest.
